# The Emerging Role of FAM171A2 in Gynecological Malignancies: Bioinformatic Insights from UCEC and Ovarian Cancer

**DOI:** 10.3390/ijms262211126

**Published:** 2025-11-18

**Authors:** Sibel Soylemez, Durmus Ayan

**Affiliations:** 1 Gazi University Life Sciences Application and Research Center, Ankara 06560, Türkiye; 2 Medical Laboratory Techniques Program, Department of Medical Services and Techniques, Gazi University Vocational School of Health Services, Ankara 06560, Türkiye; 3 Medical Biochemistry, Faculty of Medicine, Nigde Omer Halisdemir University, Nigde 51200, Türkiye; 4 Medical Biochemistry, Nigde Omer Halisdemir University Research and Training Hospital, Nigde 51200, Türkiye

**Keywords:** *FAM171A2* gene expression, gynecologic neoplasms, ovarian serous cystadenocarcinoma, uterine corpus endometrial carcinoma (UCEC), transmembrane signaling proteins, microRNA–lncRNA interaction network, post-transcriptional gene regulation, extracellular vesicle biology, tumor microenvironment modulation, bioinformatics and multi-omics analysis

## Abstract

The *FAM171A2* gene encodes a transmembrane protein that is not well characterized but is implicated in signaling, vesicle trafficking, and interactions with the extracellular matrix. Its specific role in gynecologic malignancies has yet to be defined. To our knowledge, this is the first systematic study to comprehensively assess FAM171A2 expression, clinical relevance, and molecular network interactions in gynecologic malignancies. We employed an integrative approach utilizing multi-platform transcriptomic and proteomic resources—GEPIA2, TNMplot, TIMER2, UALCAN, KM-plotter, Human Protein Atlas (HPA), Gene Expression Omnibus (GEO), STRING, TargetScan, and ENCORI—to comprehensively profile *FAM171A2* expression, its clinicopathologic correlations, survival associations, predicted interaction networks, and post-transcriptional regulation in ovarian cancer (OV) and uterine corpus endometrial carcinoma (UCEC). Immunohistochemical analysis from the HPA indicated low or undetectable levels of the FAM171A2 protein in OV and UCEC. In contrast, RNA sequencing analyses demonstrated upregulated mRNA expression in OV and a modest, non-significant increase in UCEC compared to normal tissues. Pan-cancer screening using TNMplot and TIMER2 revealed elevated expression in gynecologic tumors relative to most other cancer types. In OV, UALCAN analysis identified associations with demographic and molecular characteristics, such as increased expression in TP53-mutant tumors, while trends related to stage and grade were minimal. Similarly, stratifications in UCEC suggested modulation by race, body mass index (BMI), and menopausal status rather than stage. Survival analyses using KM-plotter showed no significant association with overall survival in either type of cancer. TargetScan predicted 211 microRNAs potentially targeting FAM171A2, and ENCORI correlations supported tumor-type-specific post-transcriptional regulation: in OV, negative correlations were observed with miR-15b-5p, miR-16-5p, and miR-497-5p, along with long non-coding RNA (lncRNA) effects, including positive correlations with BACE1-AS and negative correlations with PVT1 and UCA1. In UCEC, significant negative correlations were found with LINC00582, LINC-ROR, MEG3, NEAT1, and SNHG12. STRING network analysis suggested two modules associated with *FAM171A2*: a neuronal/synaptic cluster, exemplified by *NPTX1*, and an immune/transcriptional cluster, exemplified by *ZNF696*. Validation using the GEO showed mixed results: two UCEC datasets were non-significant, whereas one OV cohort (GSE36368) exhibited higher tumor expression. *FAM171A2* demonstrates context-dependent expressions that are modulated post-transcriptionally in gynecologic cancers. While it is not independently prognostic, it may serve as a molecular hub at the intersection of neuronal and immune pathways, warranting further mechanistic investigations and exploration as a panel-based biomarker.

## 1. Introduction

Gynecological malignancies, including ovarian and endometrial cancers, constitute a significant global health burden for women. These malignancies are often characterized by intricate molecular landscapes and present substantial challenges in treatment due to drug resistance and the widespread cytotoxicity associated with conventional therapies [1]. The urgent demand for innovative, targeted therapeutic approaches necessitates a comprehensive understanding of the molecular mechanisms driving these various cancers [2]. This unmet clinical need has catalyzed extensive research into precision medicine strategies, which utilize advanced genomic and transcriptomic profiling to discern unique molecular signatures and actionable targets within individual tumors [3]. The *FAM171* family, consisting of *FAM171A1, FAM171A2*, and *FAM171B*, represents a group of type-I transmembrane cell surface proteins that facilitate cell–cell interactions, adhesion, and signaling. Notably, *FAM171A1* has been identified as a potential oncogene that influences actin cytoskeletal organization, thereby affecting cell morphology and the invasive behavior of tumor cells [4,5,6,7], exhibiting aberrant expression and functional involvement in various cancers, including glioma and hepatocellular carcinoma [8]. *FAM171A2* is expressed in various human tissues and exhibits significant enrichment in the brain, which aligns with its potential role in vesicle-mediated neuronal communication [9,10,11]. Considering the conserved domain architecture and partial sequence homology within the *FAM171* family, it is plausible to hypothesize that *FAM171A2* may also be involved in tumorigenic processes, potentially through related pathways such as cell adhesion, migration, or extracellular vesicle-mediated signaling. Although direct experimental evidence for *FAM171A2* is currently limited, transcriptomic profiling from large-scale cancer datasets (e.g., TCGA) indicates differential expression patterns across multiple tumor types, further supporting its potential role in cancer pathogenesis [10,12]. Genetic evidence implicating *FAM171A2* in neurodegenerative disorders such as Alzheimer’s disease and Parkinson’s disease indicates its involvement in pathways that regulate cellular stress, vesicular transport, and intercellular signaling [13]. These biological processes are essential for the initiation and progression of cancer [14]. Moreover, the processes of tumor cell migration, adhesion, and microenvironmental remodeling are integral to cancer progression [14]. Therefore, although *FAM171A2* has not been previously characterized in the context of cancer, its structural attributes and functional similarities to signaling and adhesion molecules offer a biologically plausible rationale for its investigation in gynecologic malignancies. In these malignancies, dysregulated communication and vesicle dynamics are critical hallmarks of tumor behavior. In uterine corpus endometrial carcinoma (UCEC), aberrant signaling pathways such as PI3K/AKT/mTOR, Wnt/β-catenin, and NF-κB are frequently implicated [15,16,17]. The findings suggest that dysregulation of *FAM171A2* may integrate into inflammatory networks, potentially affecting the aggressiveness of endometrial tumors [18]. In ovarian cancer (OV), where late-stage diagnosis and chemoresistance present significant challenges, *FAM171A2* has been identified as a potential molecule associated with the extracellular matrix (ECM). The ECM is a dynamic structure that regulates tissue integrity and cellular behavior; however, its dysregulation may be intricately linked to the tumor development, invasion, and dissemination observed in OV [19]. The expression profile of *FAM171A2* may serve as a novel biomarker for distinguishing molecular subtypes and predicting patient survival.

In light of the absence of previous research on FAM171A2 within oncological frameworks, investigating its transcriptional and post-transcriptional regulation could yield novel insights into tumor biology. Consequently, we conducted an extensive in silico analysis, integrating various transcriptomic and proteomic databases to assess *FAM171A2* expression, its clinicopathological significance, and its regulatory interactions with miRNAs and lncRNAs in OV and UCEC Importantly, this study provides the first integrated, multi-dataset analysis of *FAM171A2* in ovarian and endometrial cancer, establishing a foundation for future mechanistic and translational investigations, with the objective of elucidating its potential role in tumor signaling networks and evaluating its viability as a candidate biomarker or therapeutic target.

## 2. Results

### 2.1. Expression Patterns of the Gene Across OV and UCEC

**Figure 1:**
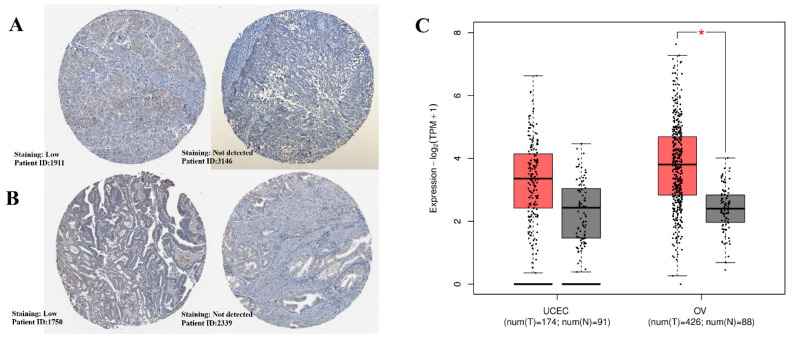
Representative immunohistochemistry (IHC) images showing low or undetectable FAM171A2 protein expression in ovarian cancer (OV, (**A**)) and uterine corpus endometrial carcinoma (UCEC), (**B**). (Scale bar = 200 µm). (**C**) Boxplot analysis of RNA-seq data demonstrates significant upregulation of FAM171A2 mRNA in OV, whereas no statistically significant alteration was detected in UCEC compared with normal tissues. Tumor samples are shown in red and normal tissues in gray. Statistically significant differences are indicated by red asterisks (*p* < 0.05). Adjusted *p*-values were calculated using the Benjamini–Hochberg FDR correction to control for multiple testing. Abbreviations: IHC, immunohistochemistry; RNA-seq, RNA sequencing; mRNA, messenger RNA; OV, ovarian cancer; UCEC, uterine corpus endometrial carcinoma.

Immunohistochemistry (IHC) analysis of patient samples from the Human Protein Atlas demonstrated that FAM171A2 protein expression was either low or undetectable in both OV and UCEC tissues (Figure 1A,B). Representative images illustrate low cytoplasmic staining or complete absence of detectable signal, suggesting minimal protein-level expression in tumor tissues.

In contrast, transcriptomic analysis revealed differential patterns between tumor and normal tissues (Figure 1C). In OV samples, FAM171A2 mRNA levels were significantly upregulated compared with normal ovarian tissues (*p* < 0.05), while no statistically significant alteration was detected in UCEC. This discrepancy between protein and RNA levels suggests potential post-transcriptional regulation or protein stability mechanisms influencing *FAM171A2* expression in gynecological malignancies.

### 2.2. Comparative Expression Analysis Across Cancer Types via TNMplot

**Figure 2:**
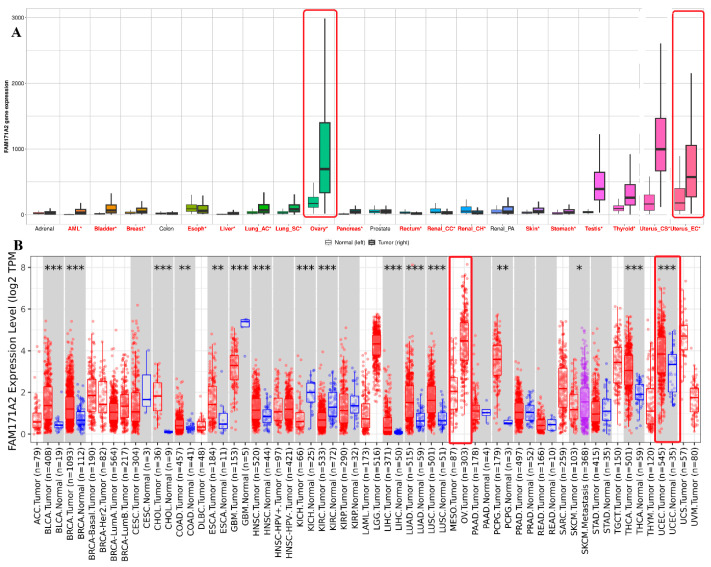
(**A**) Pan-cancer analysis of *FAM171A2* gene expression using the TNMplot platform. Expression levels are shown in normal (left) and tumor (right) tissues across multiple cancer types. Red boxes highlight ovarian cancer (OV), and uterine corpus endometrial carcinoma (UCEC), where tumor tissues exhibit notably higher expression compared with normal controls. (**B**) Differential expression of *FAM171A2* between tumor and normal tissues across multiple cancer types using TIMER2 database. Boxplots represent log2-transformed TPM values of *FAM171A2* expression in TCGA tumor samples (red) compared to corresponding normal tissues (blue). Each dot indicates an individual sample. Statistical significance was calculated using Wilcoxon test (* *p* < 0.05; ** *p* < 0.01; *** *p* < 0.001). Adjusted *p*-values were calculated using the Benjamini–Hochberg FDR correction to control for multiple testing. Abbreviations: ACC, Adrenocortical carcinoma; BLCA, Bladder urothelial carcinoma; BRCA, Breast invasive carcinoma; CESC, Cervical squamous cell carcinoma and endocervical adenocarcinoma; CHOL, Cholangiocarcinoma; COAD, Colon adenocarcinoma; DLBC, Lymphoid neoplasm diffuse large B-cell lymphoma; ESCA, Esophageal carcinoma; GBM, Glioblastoma multiforme; HNSC, Head and neck squamous cell carcinoma; KICH, Kidney chromophobe; KIRC, Kidney renal clear cell carcinoma; KIRP, Kidney renal papillary cell carcinoma; LIHC, Liver hepatocellular carcinoma; LUAD, Lung adenocarcinoma; LUSC, Lung squamous cell carcinoma; MESO, Mesothelioma; PAAD, Pancreatic adenocarcinoma; PCPG, Pheochromocytoma and paraganglioma; PRAD, Prostate adenocarcinoma; READ, Rectum adenocarcinoma; SARC, Sarcoma; SKCM, Skin cutaneous melanoma; STAD, Stomach adenocarcinoma; TGCT, Testicular germ cell tumor; THCA, Thyroid carcinoma; THYM, Thymoma; UCEC, Uterine corpus endometrial carcinoma; UCS, Uterine carcinosarcoma; UVM, Uveal melanoma, TPM, transcripts per million.

Pan-cancer analysis using the TNMplot platform revealed variable expression patterns of *FAM171A2* across malignancies. Markedly higher expression in tumor tissues compared with normal counterparts was observed in OV and UCEC. An increase was also noted in other cancer types in tumor tissues compared with normal (for example, testicular, acute myeloid leukemia, bladder cancer, lung cancer, pancreatic cancer and thyroid cancers). A decrease was noted in renal tumors (CC, CH, PA subtypes) (Figure 2A).

TIMER2 analysis revealed that *FAM171A2* expression was significantly upregulated in several cancer types. Notably, strong overexpression was observed in UCEC, Thyroid carcinoma, liver hepatocellular carcinoma (LIHC), and lung adenocarcinoma (LUAD) (*p* < 0.001) (Figure 2B). These findings suggest that *FAM171A2* may play a biologically relevant role particularly in gynecological and gastrointestinal malignancies. Overall, the most distinct differences were restricted to gynecological malignancies, while the majority of other cancer types demonstrated comparable expression profiles.

### 2.3. Comparative Expression Analysis Across Different Clinicopathological Variables via UALCAN

#### 2.3.1. Comparative Expression Analysis of OV

**Figure 3:**
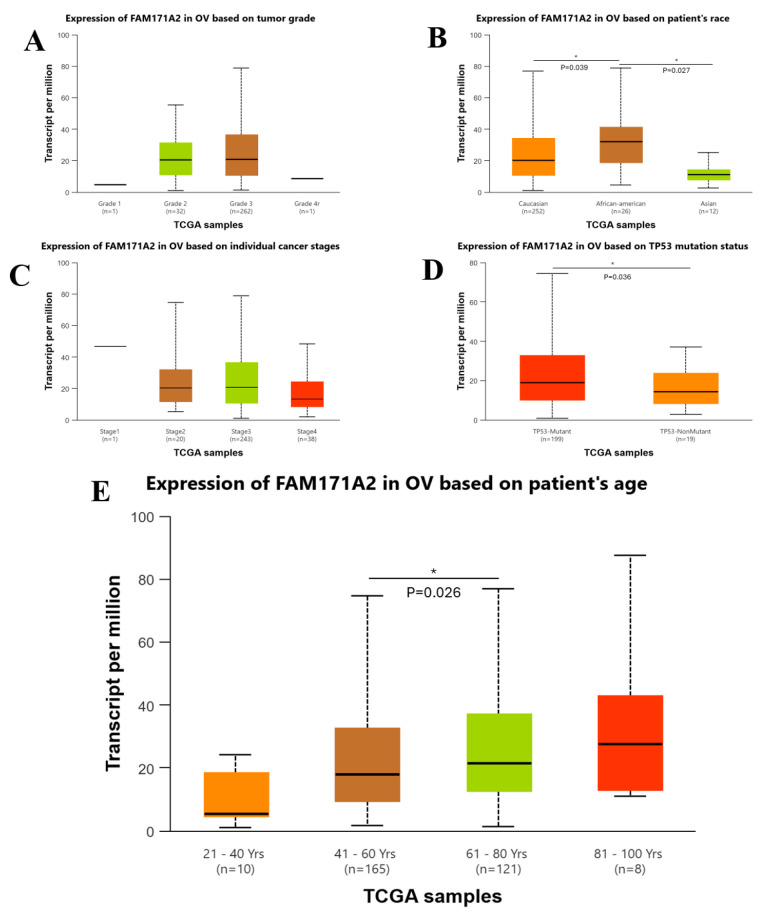
Expression of *FAM171A2* in ovarian cancer (OV) across clinicopathological variables based on TCGA samples. (**A**) Expression according to tumor grade (G1–G4). (**B**) Expression based on patient race (Caucasian, African-American, and Asian). (**C**) Expression according to individual cancer stages (Stage I–IV). (**D**) Expression in relation to TP53 mutation status (mutant vs. non-mutant). (**E**) Expression based on patient age groups (21–40, 41–60, 61–80, 81–100 years). Statistical comparisons were performed using standard methods, and significant *p*-values are indicated in the plots (* *p* < 0.05). Adjusted *p*-values were calculated using the Benjamini–Hochberg FDR correction to control for multiple testing. Abbreviations: OV, ovarian cancer; TCGA, The Cancer Genome Atlas; TP53, tumor protein 53.

The expression of *FAM171A2* in OV was evaluated across different clinicopathological variables using TCGA samples. No significant differences were observed in relation to tumor grade or clinical stage. However, analysis based on patient characteristics revealed variability. Expression levels were significantly higher in African-American patients compared to Caucasian (*p* = 0.039) and Asian (*p* = 0.027) patients. In addition, patients aged 41–60 years showed significantly higher expression compared to those aged 21–40 years (*p* = 0.026), while expression levels in older age groups remained variable. These findings suggest that *FAM171A2* expression in OV may be influenced by demographic factors such as race and age, but not by tumor grade or stage. *FAM171A2* expression was significantly higher in tumors with TP53 mutations compared to non-mutant cases (*p* = 0.036). The median expression level was elevated in the TP53-mutant group, whereas lower transcript levels were observed in TP53 non-mutant tumors. These findings indicate a potential association between TP53 mutation status and increased *FAM171A2* expression in OV (Figure 3).

#### 2.3.2. Comparative Expression Analysis of UCEC

**Figure 4:**
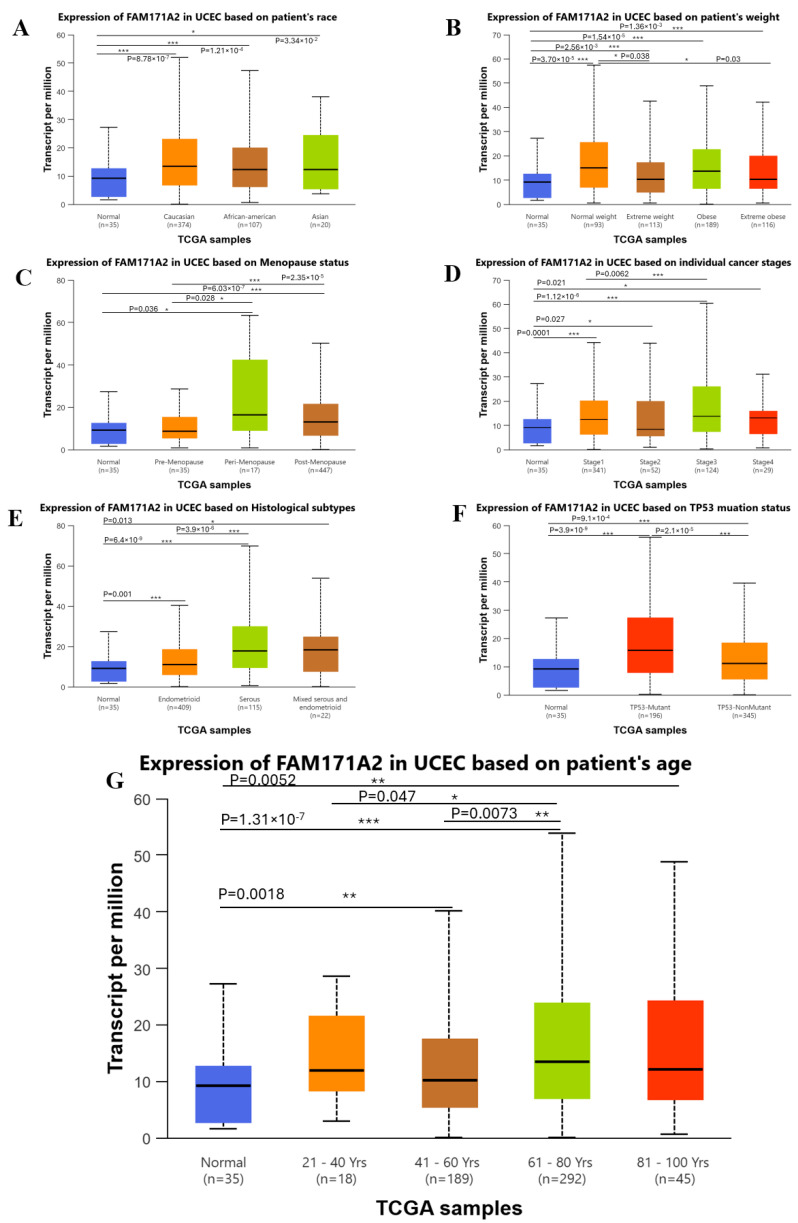
Expression of *FAM171A2* in UCEC (TCGA RNA-seq) across clinicopathological and demographic variables. Box-and-whisker plots show TPM values (boxes = median and IQR; whiskers = 1.5 × IQR). Sample sizes (n) are indicated on the x-axes. *p*-values for pairwise comparisons are displayed above brackets (*, **, *** denote *p* < 0.05, *p* < 0.01, *p* < 0.001, respectively). Adjusted *p*-values were calculated using the Benjamini–Hochberg FDR correction to control for multiple testing. (**A**) Patient race (Normal, Caucasian, African-American, and Asian), (**B**) Body weight/BMI category (Normal, Normal weight, Overweight, Obese, Extreme obese), (**C**) Menopausal status (Normal, Pre-menopause, Peri-menopause, Post-menopause), (**D**) Clinical stage (Normal, Stage I–IV), (**E**) Histological subtype (Normal, Endometrioid, Serous, Mixed serous & endometrioid), (**F**) TP53 mutation status (Normal, TP53-Mutant, TP53-NonMutant), (**G**) Age groups (Normal, 21–40, 41–60, 61–80, 81–100 years). Abbreviations: UCEC, uterine corpus endometrial carcinoma; TCGA, The Cancer Genome Atlas; RNA-seq, RNA sequencing; BMI, body mass index; TPM, transcripts per million; TP53, tumor protein 53; IQR, interquartile range.

Overall, tumor tissues showed higher *FAM171A2* expression than normal endometrium across strata. By race, expression was highest in African-American patients and lower in Caucasian and Asian cohorts, with significant pairwise differences (Caucasian vs. African-American: *p* = 8.78 × 10^−7^; African-American vs. Asian: *p* = 1.21 × 10^−4^; Caucasian vs. Asian: *p* = 3.34 × 10^−2^). With respect to body weight, all tumor BMI groups (normal weight, overweight, obese, extreme obese) were higher than normal tissue (several very small *p*-values annotated in the plot) and selected within-tumor comparisons were significant (normal weight vs. extreme weight: *p* = 0.038; normal weight vs. extreme-obese: *p* = 0.030; normal vs. normal weight: *p* = 3.70 × 10^−5^; normal vs. extreme obese: *p* = 2.56 × 10^−3^; normal vs. obese: *p* = 1.54 × 10^−5^; normal vs. extreme obese: *p* = 1.36 × 10^−3^). Menopause status was associated with variability, with the peri-menopausal group showing the highest median; several pairwise tests reached significance (e.g., normal vs. pre-menopause: *p* = 0.038; pre- vs. peri-menopause: *p* = 0.028; normal vs. post-menopause: *p* = 6.03 × 10^−7^; pre- vs. peri-menopause: *p* = 2.35 × 10^−5^). *FAM171A2* did not display a monotonic gradient across clinical stages, but each stage exceeded normal tissue (normal vs. stage I: *p* = 0.00001; normal vs. stage II: *p* = 0.027; normal vs. stage III: *p* = 1.12 × 10^−6^; normal vs. stage IV: *p* = 0.021; stage I vs. stage III: *p* = 0.0062). Across histologic subtypes, serous tumors were highest, mixed intermediate, and endometrioid lower, with significant differences (normal vs. endometrioid: *p* = 0.001; normal vs. serous: *p* = 6.4 × 10^−9^; endometrioid vs. serous: *p* = 3.9 × 10^−6^; normal vs. mixed endometrioid and serous: *p* = 0.013). Tumors with TP53 mutations showed higher expression than non-mutant cases; the panel also indicates normal vs. TP53-mutant: *p* = 3.9 × 10^−9^, normal vs. non-mutant: *p* = 2.1 × 10^−4^, and mutant vs. non-mutant: *p* = 2.1 × 10^−5^. By age, expression increased in older groups with several significant contrasts (normal vs. 41–60 years: *p* = 0.0018; normal vs. 61–80 years: *p* = 1.31 × 10^−7^; 21–40 vs. 61–80: *p* = 0.047; 41–60 vs. 61–80: *p* = 0.0073; normal vs. 81–100: *p* = 0.0052). Collectively, these data suggest that demographic and biological factors (race, BMI, menopause, histology, age, TP53 status)—rather than clinical stage—primarily modulate *FAM171A2* expression in UCEC (Figure 4).

### 2.4. Kaplan–Meier Survival Analysis of FAM171A2 in UCEC and OV

**Figure 5:**
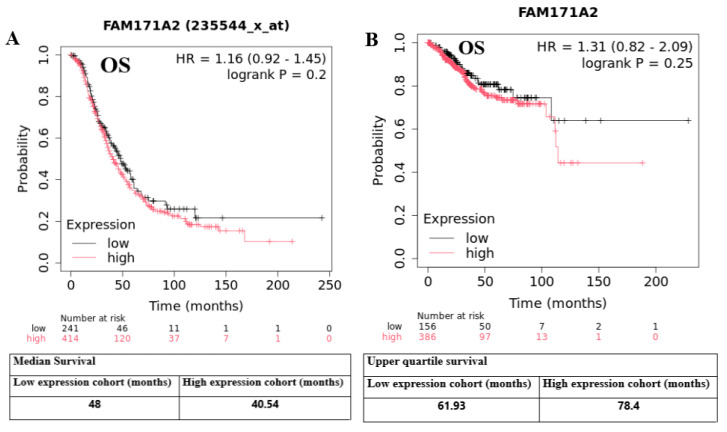
Kaplan–Meier plots of overall survival (OS) stratified by *FAM171A2* expression. Curves compare low (gray) vs. high (red) expression cohorts; tick marks indicate censored observations. The *x*-axis shows time (months) and the *y*-axis shows survival probability. Numbers at risk are given below each plot; summary survival metrics are reported in the tables under the panels. (**A**) Ovarian cancer (OV; KMplot, probe 235544_x_at). Hazard ratio (HR) 1.16 (95% CI, 0.92–1.45); log-rank *p* = 0.20. Median OS: 48 months (low) vs. 40.54 months (high). (**B**) Uterine corpus endometrial carcinoma (UCEC; KMplot). HR 1.31 (95% CI, 0.82–2.09); log-rank *p* = 0.25. Upper-quartile survival: 61.93 months (low) vs. 78.4 months (high). Abbreviations: OV, ovarian cancer; UCEC, uterine corpus endometrial carcinoma; OS, overall survival; HR, hazard ratio; CI, confidence interval.

High *FAM171A2* expression was not significantly associated with overall survival (HR = 1.16, 95% CI: 0.92–1.45; log-rank *p* = 0.20) in OV. The median OS was 40.54 months in the high-expression cohort versus 48 months in the low-expression cohort, indicating only a non-significant shorter OS with higher expression. Baseline numbers-at-risk were imbalanced (high n = 414, low n = 241), which may reduce precision (Figure 5A). No statistically significant difference in OS was observed in UCEC (HR = 1.31, 95% CI: 0.82–2.09; log-rank *p* = 0.25). The upper-quartile survival (KMplot metric) was 78.4 months for the high-expression group and 61.93 months for the low-expression group; however, this early-time summary contrasts with the HR (>1.0) and is not significant, likely to reflect event-time distribution and cohort imbalance (high n = 386, low n = 156) (Figure 5B). Across both cohorts, *FAM171A2* expression showed no significant association with OS in either OV or UCEC.

### 2.5. Network Visualization of FAM171A2 and Its Associated miRNAs

**Figure 6:**
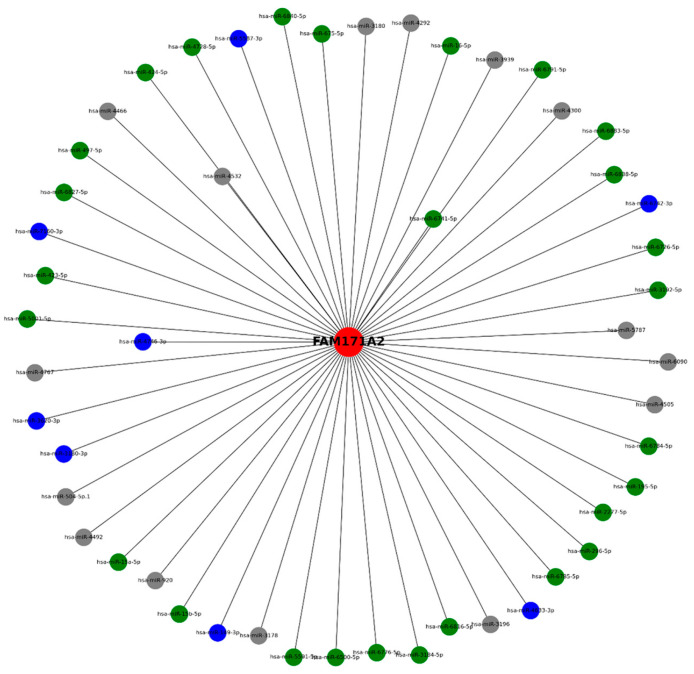
Network diagram of *FAM171A2* and its top 50 associated miRNAs. The central FAM171A2 gene is indicated in red, while surrounding miRNAs are colored according to their strand origin: 3p (blue), 5p (green), and unannotated/other variants (gray). Abbreviations: miRNA, microRNA.

As a result of the analysis performed with TargetScan 8.0, all *FAM171A2*-associated miRNAs are shown in Table 1. This network diagram depicts the *FAM171A2* gene at the center, surrounded by the top 50 associated miRNAs according to context++ score percentile (Figure 6). The nodes are color-coded according to their strand origin:

- Green nodes represent miRNAs derived from the 5p arm.
- Blue nodes represent miRNAs derived from the 3p arm.
- Gray nodes correspond to variants or miRNAs without explicit arm annotation.
- Red node highlights the central *FAM171A2* gene.

**Table 1:** All miRNAs associated with *FAM171A2* (n = 211).

TargetScan8.0
hsa-miR-6838-5p, hsa-miR-15b-5p, hsa-miR-497-5p, hsa-miR-16-5p, hsa-miR-424-5p, hsa-miR-195-5p, hsa-miR-15a-5p, hsa-miR-4746-3p, hsa-miR-6816-5p, hsa-miR-3196, hsa-miR-3180-3p, hsa-miR-3180, hsa-miR-423-5p, hsa-miR-3184-5p, hsa-miR-6785-5p, hsa-miR-4728-5p, hsa-miR-6883-5p, hsa-miR-149-3p, hsa-miR-2277-5p, hsa-miR-4767, hsa-miR-4466, hsa-miR-675-5p, hsa-miR-6741-5p, hsa-miR-6776-5p, hsa-miR-6742-3p, hsa-miR-6791-5p, hsa-miR-4292, hsa-miR-504-5p.1, hsa-miR-3620-3p, hsa-miR-3178, hsa-miR-6784-5p, hsa-miR-4532, hsa-miR-5587-3p, hsa-miR-6840-5p, hsa-miR-296-5p, hsa-miR-296-5p, hsa-miR-7160-3p, hsa-miR-3939, hsa-miR-4633-3p, hsa-miR-6500-5p, hsa-miR-6726-5p, hsa-miR-5591-5p, hsa-miR-920, hsa-miR-4300, hsa-miR-6090, hsa-miR-6827-5p, hsa-miR-3192-5p, hsa-miR-4505, hsa-miR-5787, hsa-miR-4492, hsa-miR-5001-5p, hsa-miR-4498, hsa-miR-762, hsa-miR-185-3p, hsa-miR-4489, hsa-miR-4283, hsa-miR-6852-5p, hsa-miR-661, hsa-miR-7107-5p, hsa-miR-1234-3p, hsa-miR-6850-3p, hsa-miR-6892-3p, hsa-miR-4749-3p, hsa-miR-296-5p, hsa-miR-6724-5p, hsa-miR-6773-5p, hsa-miR-939-3p, hsa-miR-4292, hsa-miR-6791-5p, hsa-miR-331-3p, hsa-miR-210-5p, hsa-miR-3922-5p, hsa-miR-5695, hsa-miR-6736-3p, hsa-miR-29c-3p, hsa-miR-29b-3p, hsa-miR-29a-3p, hsa-miR-3065-3p, hsa-miR-551b-3p, hsa-miR-551a, hsa-miR-4696, hsa-miR-6841-3p, hsa-miR-4780, hsa-miR-6780b-3p, hsa-miR-623, hsa-miR-6768-5p, hsa-miR-3166, hsa-miR-6511a-5p, hsa-miR-1910-3p, hsa-miR-1827, hsa-miR-3612, hsa-miR-650, hsa-miR-4443, hsa-miR-6515-5p, hsa-miR-432-5p, hsa-miR-4707-5p, hsa-miR-6763-3p, hsa-miR-5587-3p, hsa-miR-6749-3p, hsa-miR-5193, hsa-miR-4667-3p, hsa-miR-6887-3p, hsa-miR-6859-3p, hsa-miR-711, hsa-miR-4638-3p, hsa-miR-422a, hsa-miR-378f, hsa-miR-378i, hsa-miR-378c, hsa-miR-378e, hsa-miR-378a-3p, hsa-miR-378d, hsa-miR-378h, hsa-miR-378b, hsa-miR-6835-5p, hsa-miR-6803-5p, hsa-miR-6751-5p, hsa-miR-6752-5p, hsa-miR-6842-5p, hsa-miR-7110-5p, hsa-miR-4447, hsa-miR-4472, hsa-miR-1306-5p, hsa-miR-4707-5p, hsa-miR-6763-3p, hsa-miR-5587-3p, hsa-miR-6749-3p, hsa-miR-5193, hsa-miR-4667-3p, hsa-miR-4290, hsa-miR-4687-5p, hsa-miR-361-3p, hsa-miR-3679-3p, hsa-miR-2115-5p, hsa-miR-7162-5p, hsa-miR-516a-3p, hsa-miR-516b-3p, hsa-miR-4269, hsa-miR-6715b-5p, hsa-miR-6768-5p, hsa-miR-4672, hsa-miR-3618, hsa-miR-4691-3p, hsa-miR-6856-3p, hsa-let-7a-2-3p, hsa-let-7g-3p, hsa-miR-7159-3p, hsa-miR-4482-3p, hsa-miR-3160-5p, hsa-miR-188-3p, hsa-miR-3156-3p, hsa-miR-1260b, hsa-miR-1260a, hsa-miR-3160-5p, hsa-miR-6893-3p, hsa-miR-370-3p, hsa-miR-1976, hsa-miR-660-3p, hsa-miR-4667-3p, hsa-miR-6887-3p, hsa-miR-6802-3p, hsa-miR-6879-3p, hsa-miR-5589-5p, hsa-miR-4505, hsa-miR-5787, hsa-miR-6884-5p, hsa-miR-485-5p, hsa-miR-3975, hsa-miR-2467-5p, hsa-miR-3188, hsa-miR-4649-3p, hsa-miR-7160-5p, hsa-miR-646, hsa-miR-503-5p, hsa-miR-497-5p, hsa-miR-424-5p, hsa-miR-6838-5p, hsa-miR-15a-5p, hsa-miR-15b-5p, hsa-miR-4524b-5p, hsa-miR-4524a-5p, hsa-miR-195-5p, hsa-miR-16-5p, hsa-miR-4704-5p, hsa-miR-216b-3p, hsa-miR-342-3p, hsa-miR-4687-5p, hsa-miR-7977, hsa-miR-6734-3p, hsa-miR-5088-3p, hsa-miR-4685-3p, hsa-miR-4287, hsa-miR-6887-3p, hsa-miR-4313, hsa-miR-3133, hsa-miR-615-5p, hsa-miR-1915-3p, hsa-miR-6764-5p, hsa-miR-4726-3p, hsa-miR-6840-3p, hsa-miR-6887-3p, hsa-miR-6795-3p, hsa-miR-6826-3p, hsa-miR-6887-3p, hsa-miR-4640-3p, hsa-miR-6871-3p, hsa-miR-3065-3p, hsa-miR-545-3p, hsa-miR-8086, hsa-miR-664b-5p, hsa-miR-1273f, hsa-miR-4756-3p, hsa-miR-3913-3p, hsa-miR-489-3p, hsa-miR-4504, hsa-miR-542-3p, hsa-miR-146b-3p, hsa-miR-6779-3p, hsa-miR-1226-3p, hsa-miR-4691-3p, hsa-miR-7977, hsa-miR-4433a-5p, hsa-miR-4433b-5p, hsa-miR-2355-5p, hsa-miR-5588-3p, hsa-miR-2114-5p, hsa-miR-554, hsa-miR-4640-3p, hsa-miR-6798-3p, hsa-miR-323a-3p, hsa-miR-130a-5p, hsa-miR-23a-3p, hsa-miR-23c, hsa-miR-23b-3p, hsa-miR-4999-5p, hsa-miR-6882-3p, hsa-miR-6083, hsa-miR-4328, hsa-miR-4733-3p, hsa-miR-1226-3p, hsa-miR-6511b-3p, hsa-miR-6511a-3p, hsa-miR-3150a-5p, hsa-miR-3150b-5p

Such visualization provides an overview of the potential post-transcriptional regulatory network of *FAM171A2*, illustrating how diverse miRNA variants (3p/5p) may interact with and modulate this gene. The distribution of miRNAs across the two arms may further suggest functional heterogeneity in gene regulation.

### 2.6. Correlation Analysis of FAM171A2 Expression with Selected miRNAs in Ovarian and Endometrial Cancers Using ENCORI

**Figure 7:**
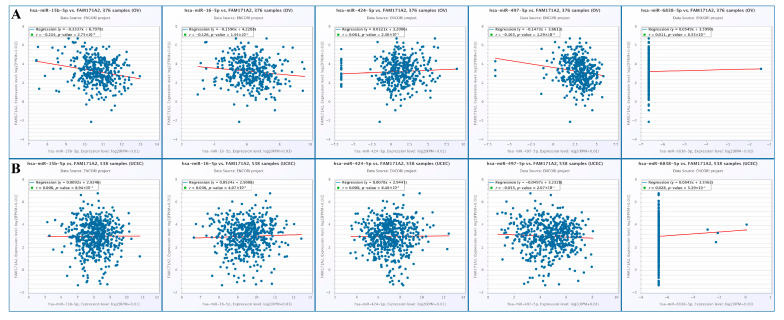
Scatter plot analysis of *FAM171A2* and selected miRNAs (Top 5 miRNAs) across TCGA OV (**A**) and UCEC (**B**) cancer cohorts. Abbreviations: miRNA, microRNA; mRNA, messenger RNA; OV, ovarian cancer; UCEC, uterine corpus endometrial carcinoma; TCGA, The Cancer Genome Atlas.

Scatter plots illustrate the correlations between *FAM171A2* mRNA levels and the indicated miRNAs (hsa-miR-15b-5p, hsa-miR-16-5p, hsa-miR-424-5p, hsa-miR-497-5p, and hsa-miR-6838-5p) in OV samples (n = 376). Significant negative correlations were observed for miR-15b-5p (r = – 0.214, *p* = 2.75 × 10^−5^), miR-16-5p (r = – 0.126, *p* = 0.014), and miR-497-5p (r = – 0.165, *p* = 0.013), suggesting potential post-transcriptional suppression of *FAM171A2* by these miRNAs in O (Figure 7A). The same miRNAs were analyzed in UCEC samples (n = 538); however, none of the correlations reached statistical significance (*p* > 0.05) (Figure 7B).

### 2.7. Comparative Network Analysis of FAM171A2: STRING

**Figure 8:**
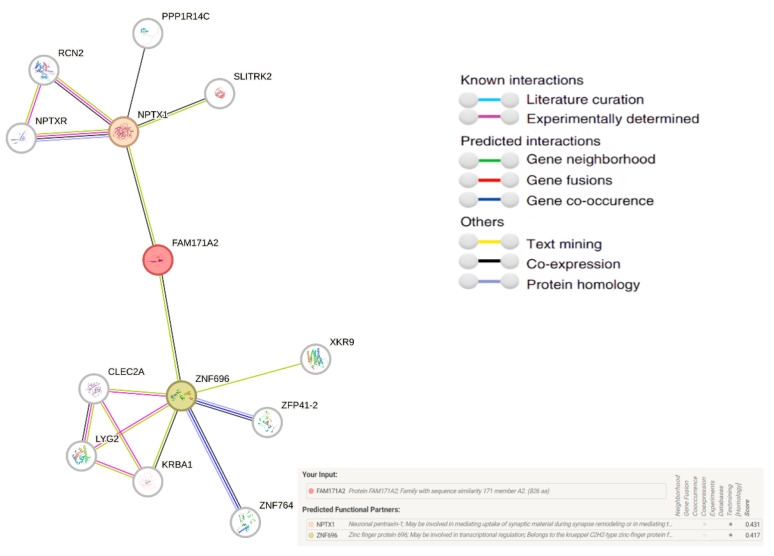
Predicted protein–protein interaction (PPI) network of FAM171A2 generated using STRING database. FAM171A2 interacts with two main clusters: (i) a neuronal module (NPTX1-centered) associated with synaptic remodeling, and (ii) a zinc finger/immune-related module (ZNF696-centered) linked to transcriptional regulation. Interaction sources include literature curation, experimental data, text mining, co-expression, and protein homology, as indicated by edge colors. Abbreviations: PPI, protein–protein interaction; STRING, Search Tool for the Retrieval of Interacting Genes/Proteins; NPTX1, neuronal pentraxin-1; ZNF696, zinc finger protein 696.

Analysis of the STRING database revealed the predicted protein–protein interaction (PPI) network of FAM171A2 (Figure 8). Two major clusters were observed around this gene: (i) a neuronal module centered on NPTX1 (neuronal pentraxin-1) and its interactors (NPTXR, RCN2, SLITRK2, PPP1R14C), which are mainly involved in synaptic remodeling and neuronal signaling; and (ii) a transcriptional/immune-related module centered on ZNF696 (zinc finger protein 696) with interactions involving ZNF764, ZFP41-2, KRBA1, LYG2, CLEC2A, and XKR9, proteins mostly implicated in transcriptional regulation and immune modulation (Figure 8). Interestingly, FAM171A2 connected with both modules, suggesting a potential dual role in neuronal pathways and transcriptional/immune processes. Given the context of gynecological malignancies, these associations may indicate unexplored functions of FAM171A2 in tumor microenvironment regulation, immune crosstalk, and possibly neuro-immune signaling within cancer biology.

### 2.8. GEO-Based Expression Analysis FAM171A2 at Across Gynecologic Cancer Cohorts

#### 2.8.1. GEO-Based Expression Analysis *FAM171A2* for UCEC

**Figure 9:**
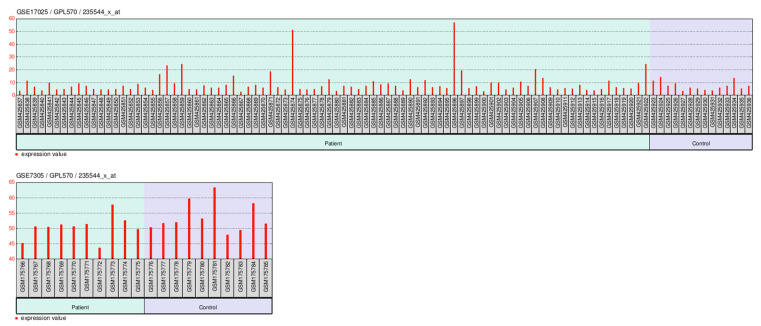
Expression of probe 235544_x_at across GEO cohorts. Boxplots show expression levels in tumor and control samples from two independent UCEC datasets (GSE17025, GSE63678). While individual datasets displayed variable trends, statistical comparisons between tumor and control groups yielded non-significant *p*-values (*p* > 0.05). Outliers indicate sample heterogeneity within cohorts. Abbreviations: GEO, Gene Expression Omnibus; UCEC, uterine corpus endometrial carcinoma.

In the UCEC cohort GSE17025, probe 235544_x_at showed overall low expression in both patients and controls, with sporadic high outliers among patients. The group comparison did not reach statistical significance (*p* > 0.05). In the UCEC cohort GSE63678, expression levels were likewise comparable between patients and controls, without consistent up- or downregulation. Statistical testing again revealed non-significant differences (*p* > 0.05) (Figure 9). Together, the two independent UCEC datasets indicate no reproducible expression change in this probe between tumor and control tissues.

#### 2.8.2. GEO-Based Expression Analysis *FAM171A2* for OV

**Figure 10:**
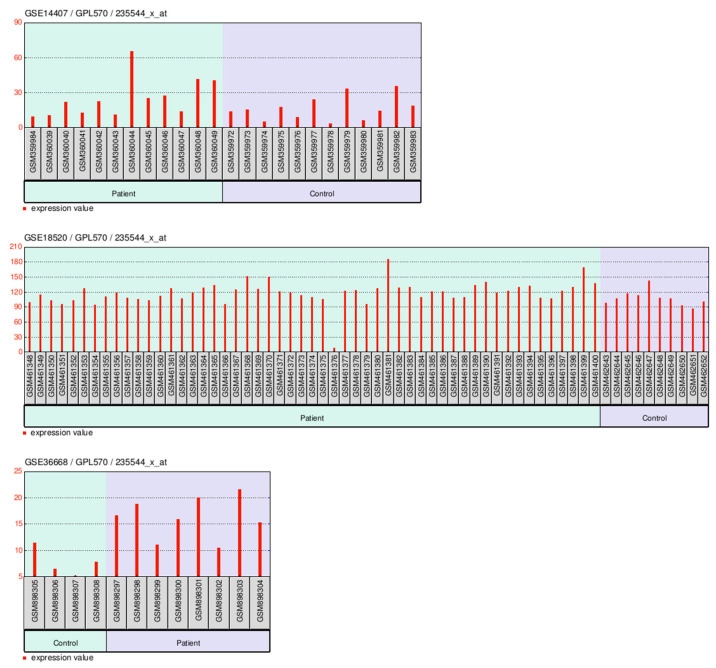
Expression of probe 235544_x_at across independent GEO OV cohorts (GSE14407, GSE18520, and GSE36368) using platform GPL570. Red bars represent normalized expression values in patient (green background) and control (purple background) groups. Abbreviations: GEO, Gene Expression Omnibus; OV, ovarian cancer.

In the OV cohorts (GSE14407, GSE18520, and GSE36368), expression of probe 235544_x_at was evaluated between tumor and control samples. GSE14407 and GSE18520 showed no statistically significant differences (*p* > 0.05), despite variable expression patterns and outliers within patient groups. In contrast, GSE36368 demonstrated significantly higher expression in tumor samples compared to controls (*p* = 0.018) (Figure 10). These findings indicate that while most OV datasets did not reveal consistent changes, one cohort suggested potential upregulation of this probe in tumor tissues.

### 2.9. LncRNAs Associated with OV and UCEC

**Figure 11:**
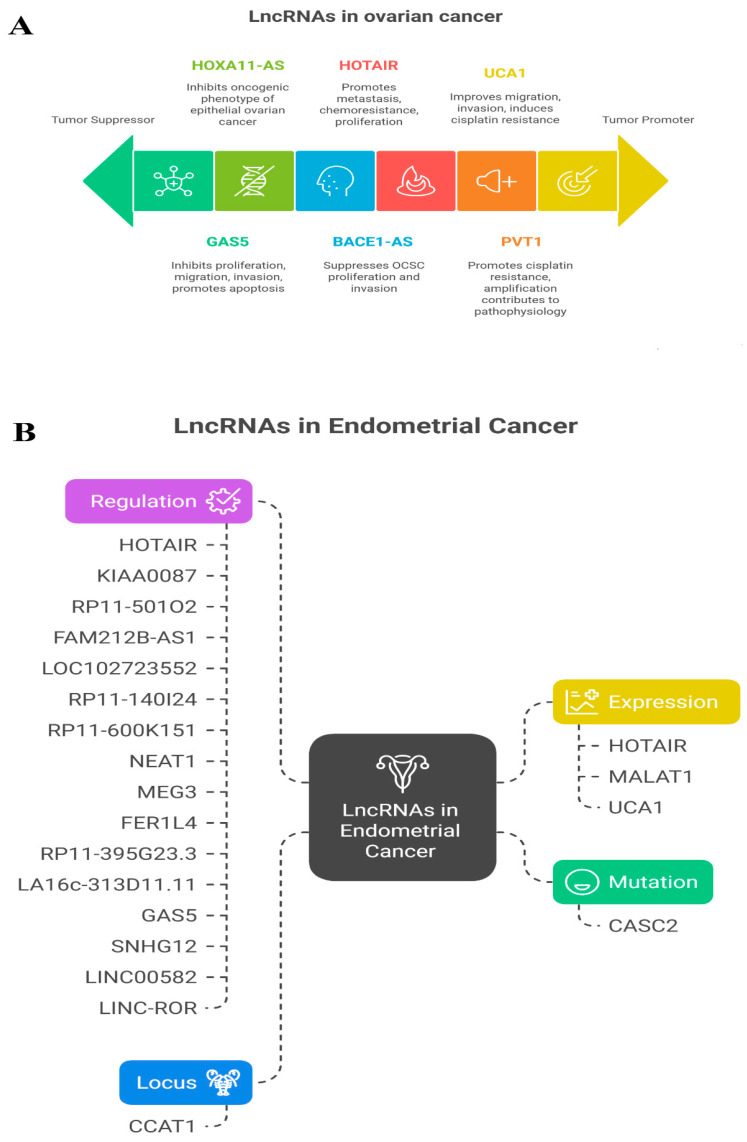
LncRNAs associated with OV (**A**) and UCEC (**B**) (from LncRNADisease database (http://www.cuilab.cn/lncrnadisease accessed on 15 September 2025). Abbreviations: lncRNA, long non-coding RNA; OV, ovarian cancer; UCEC, uterine corpus endometrial carcinoma.

LncRNAs associated with OV and UCEC are shown in Figure 11.

### 2.10. Correlation Analysis of FAM171A2 Expression with Selected lncRNAs at Across Gynecologic Cancer Cohorts

#### 2.10.1. Correlation Analysis of *FAM171A2* Expression with Selected miRNAs in OV

**Figure 12:**
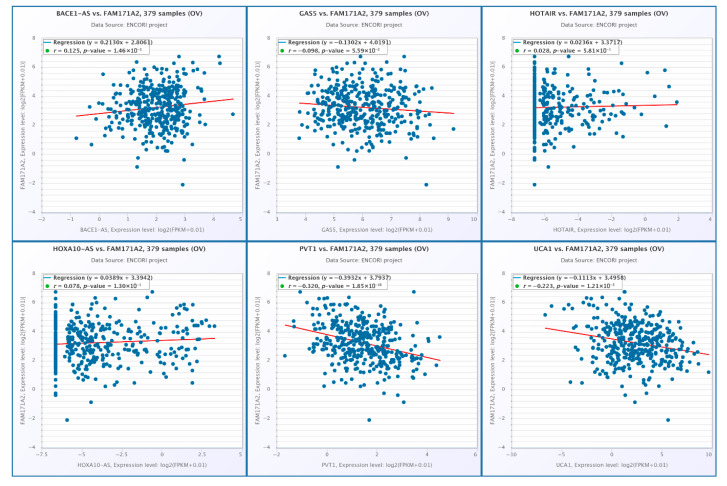
Scatter plot analysis of correlations between *FAM171A2* and selected lncRNAs in TCGA -OV. Abbreviations: lncRNA, long non-coding RNA; OV, ovarian cancer.

Scatter plots (Figure 12) show the correlations between *FAM171A2* mRNA expression and six lncRNAs (BACE1-AS, GAS5, HOTAIR, HOXA10-AS, PVT1, and UCA1) in ovarian serous cystadenocarcinoma samples (n = 379) from the ENCORI database. BACE1-AS exhibited a significant positive correlation with FAM171A2 (r = 0.213, *p* = 0.0146), while PVT1 (r = –0.320, *p* = 1.85 × 10^−10^) and UCA1 (r = –0.223, *p* = 1.21 × 10^−5^) showed significant negative correlations. No significant associations were observed for GAS5, HOTAIR, or HOXA10-AS.

#### 2.10.2. Correlation Analysis of *FAM171A2* Expression with Selected lncRNAs in UCEC

**Figure 13:**
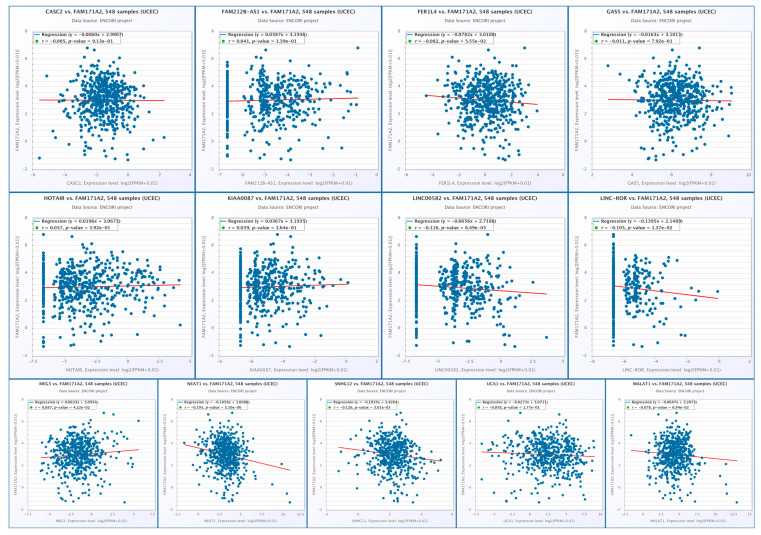
Scatter plot analysis of correlations between *FAM171A2* and selected lncRNAs in the TCGA- UCEC cohort. Abbreviations: lncRNA, long non-coding RNA; UCEC, uterine corpus endometrial carcinoma.

Correlation analyses using the ENCORI platform revealed several statistically significant negative correlations between *FAM171A2* mRNA expression and selected lncRNAs in the TCGA-UCEC cohort (n = 548). Specifically, LINC00582 (r = –0.116, *p* = 6.49 × 10^−3^), LINC-ROR (r = –0.105, *p* = 1.37 × 10^−2^), MEG3 (r = –0.084, *p* = 4.22 × 10^−2^), NEAT1 (r = –0.135, *p* = 2.56 × 10^−3^), and SNHG12 (r = –0.139, *p* = 1.74 × 10^−3^) exhibited significant inverse associations with FAM171A2. For all other lncRNAs (CASC2, FAM212B-AS1, FERI1L4, GAS5, HOTAIR, KIAA0087, MALAT1, UCA1), no significant correlation was detected as *p* > 0.05 (Figure 13).

## 3. Discussion

This study represents the first systematic examination of *FAM171A2* in gynecological malignancies, specifically focusing on OV and UCEC. Our comprehensive multi-platform analysis identified tumor-type–specific expression variations, a notable discrepancy between transcriptomic and proteomic levels, and novel interaction networks connecting *FAM171A2* to neuronal and immune pathways. These findings position *FAM171A2* as a previously unrecognized molecular node in gynecological cancers and lay the groundwork for mechanistic studies. We observed a significant upregulation of *FAM171A2* mRNA in OV, whereas no statistically significant alteration was detected in UCEC. This divergence underscores the biological heterogeneity of gynecological malignancies. OV is characterized by extensive genomic instability, EMT, and ECM remodeling, all of which contribute to metastatic spread and chemoresistance [19,20,21]. In contrast, the progression of UCEC is frequently characterized by modifications in the PI3K/AKT/mTOR and Wnt/β-catenin signaling pathways [15,16]. Our findings indicate that *FAM171A2* is more intricately involved in the EMT-driven biological processes of OV than in the canonical oncogenic pathways of UCEC, aligning with the tumor-type–specific oncogenic dependencies.

Although there is an upregulation at the RNA level, the expression of the *FAM171A2* protein was either “low or not detected” in both OV and UCEC, as demonstrated by immunohistochemistry. This RNA–protein discordance is increasingly acknowledged in cancer research and is frequently attributed to post-transcriptional repression, translational inefficiency, or protein instability [22,23]. In support of this, our TargetScan analysis identified >200 miRNAs potentially regulating *FAM171A2*, including the miR-15/16/195/497 family, well-known regulators of apoptosis, proliferation [24], and EMT in gynecological cancers [25]. These findings strongly suggest that post-transcriptional mechanisms control *FAM171A2* abundance, balancing its RNA upregulation with protein-level suppression.

Although FAM171A2 protein levels are low, the post-transcriptional regulation observed in our analysis suggests that *FAM171A2* may participate in cancer progression through RNA-based mechanisms rather than through protein abundance alone. *FAM171A2* transcripts showed strong negative correlations with the tumor-suppressor miR-15/16 family [26] and with tumor-promoting lncRNAs such as UCA1 and PVT1 [27,28]. These regulatory axes are known to drive proliferation, EMT, chemoresistance, and immune evasion in gynecologic tumors [27,28]. It is therefore plausible that *FAM171A2* functions as a competing endogenous RNA (ceRNA) [29], buffering or sequestering key miRNAs and thereby influencing oncogenic pathways even when its protein is minimally expressed. This ceRNA-like behavior, together with potential incorporation of *FAM171A2* transcripts into EV [30], may facilitate tumor–microenvironment communication and metastatic signaling. Thus, post-transcriptional dysregulation of *FAM171A2* could represent a non-canonical but biologically relevant mechanism contributing to gynecologic cancer progression.

Our analyses utilizing UALCAN have demonstrated that the expression of *FAM171A2* is influenced by demographic and molecular characteristics rather than by clinical stage or grade. Significantly, elevated expression levels were observed in African-American patients and in tumors harboring TP53 mutations. TP53 mutations are nearly ubiquitous in high-grade serous ovarian cancer (HGSC) and contribute to tumor aggressiveness, genomic instability, and resistance to therapy [31,32]. The correlation between TP53 mutation and elevated *FAM171A2* expression indicates a potential functional synergy between these molecular events. The lack of stage-specific differences suggests that *FAM171A2* deregulation may represent an early molecular alteration rather than a marker of disease progression.

Kaplan–Meier analyses demonstrated no statistically significant correlation between *FAM171A2* expression and overall survival in either OV or UCEC, although reduced OS was noted in OV. This suggests that *FAM171A2* is unlikely to function as an independent prognostic biomarker. Rather, it may be more effective as a component of multi-gene signatures or molecular panels, which frequently surpass single-gene predictors in complex malignancies [33,34,35]. Future research that integrates *FAM171A2* with EMT markers, TP53 status, or immune infiltrate profiles may enhance predictive capabilities.

From a clinical perspective, the dysregulation of *FAM171A2* may hold context-dependent significance. Its overexpression at the mRNA level, particularly in ovarian cancer, could indicate increased vesicular trafficking, cytoskeletal remodeling, or immune–stromal interaction pathways known to underlie tumor invasion, chemoresistance, and microenvironmental adaptation. Previous studies have demonstrated that *FAM171A2* is transported within EVs [36,37]. Thus, elevated *FAM171A2* transcripts may serve as a molecular indicator of aggressive tumor behavior or altered EV communication. In contrast, the overall low protein expression observed across datasets suggests that *FAM171A2* is unlikely to function as a conventional diagnostic or prognostic protein biomarker. Instead, it may provide clinical value when integrated into multi-gene signatures, ceRNA networks, or EV-associated biomarker panels to improve molecular stratification and early detection strategies in gynecologic cancers. Although *FAM171A2* did not demonstrate independent prognostic value in either OV or UCEC, this does not diminish its potential biological relevance. Accumulating evidence shows that complex malignancies such as ovarian and endometrial cancers are governed by multi-gene regulatory networks rather than single-gene drivers. Therefore, *FAM171A2* may hold greater clinical utility as part of a multi-gene signature, particularly those associated with EMT, TP53 mutation status, or extracellular matrix remodeling. Furthermore, given its predicted involvement in vesicle trafficking and RNA-based regulatory interactions, *FAM171A2* may contribute to EV-mediated communication, which plays a critical role in tumor invasion, immune crosstalk, and chemoresistance. Future mechanistic and translational studies integrating *FAM171A2* into EV cargo profiles or ceRNA network–based biomarker panels may therefore provide more clinically actionable insights than single-marker evaluation.

Our network analysis has delineated two distinct FAM171A2 modules: a neuronal cluster, centered on NPTX1, and an immune/transcriptional cluster, centered on ZNF696 [38]. The neuronal association is particularly noteworthy, as recent studies have demonstrated that tumors exploit neural pathways to facilitate growth, invasion, and perineural spread [39,40]. Although the biological role of *FAM171A2* has not yet been clearly defined, independent transcriptomic studies have reported that FAM171A2 and NPTX1 exhibit coordinated differential expression patterns (validated both at the microarray and RT-qPCR level), suggesting that these genes may be co-regulated within shared signaling networks [41]. Together with proteomic evidence showing that both proteins localize to membrane-associated structures involved in motility and structural remodeling [42], these findings provide biological plausibility for our hypothesis that FAM171A2 and NPTX1 may converge on similar pathways in gynecologic cancers.

ZNF696, a zinc finger protein located on chromosome 8q24.3, has recently been identified as a significant amplified driver gene in high-grade serous ovarian carcinoma (HGSOC). It plays a regulatory role in multiple co-expression modules associated with microtubule organization, immune activation, and Ras-mediated signaling [43]. Although the exact interaction between them remains unknown, our network analysis identified a transcriptional cluster centered on ZNF696 [44]. This observation indicates that a functional interaction between the transcriptional programs of FAM171A2 and ZNF696 might exist. Such interactions may indicate a coordinated regulation of cytoskeletal remodeling and vesicle trafficking, which are critical processes in tumor invasion and metastatic dissemination. Collectively, these findings propose that *FAM171A2* may act as a convergence point for neuronal and immune signaling in gynecological cancers, a novel concept warranting further experimental validation.

FAM171A2’s neuronal functions may have biologically relevant parallels in cancer. In the nervous system, FAM171A2 contributes to synaptic vesicle uptake and receptor-mediated fibril internalization [45]. Mechanisms that are also hijacked by cancer cells to facilitate vesicle-mediated communication, motility, and tumor microenvironment remodeling. Similar processes underpin neuroendocrine differentiation and perineural invasion, where tumor cells acquire neural-like phenotypes and interact closely with peripheral nerves to enhance survival and dissemination [46]. Furthermore, neuronal adhesion molecules and vesicle-associated proteins have been shown to regulate tumor innervation, local immune suppression, and therapy resistance/chemoresistance [47]. Thus, the neuronal characteristics of *FAM171A2* may endow gynecologic tumor cells with enhanced adaptability within neural and immune niches, providing a biologically plausible basis for its dual functional signature.

The limited upregulation of *FAM171A2* in gynecological cancers, particularly OV, as compared to other tumor types, underscores its potential as a tissue-specific biomarker. Furthermore, its anticipated role in vesicle trafficking and ECM interactions suggests a possible involvement in EV biology, which is a critical factor in metastasis, chemoresistance, and intercellular communication [48,49]. If experimentally validated, *FAM171A2* may represent a novel target for EV modulation, with potential applications in both diagnostic methodologies, such as liquid biopsy assays, and therapeutic strategies, including miRNA-based targeting and EV engineering.

In addition to transcriptional regulation, our study elucidates a sophisticated post-transcriptional network governing *FAM171A2* through interactions with miRNA and lncRNA. Among the 211 predicted miRNAs, the miR-15/16/497 family consistently emerged as negative correlations of *FAM171A2* in OV. This miRNA cluster operates as a canonical tumor-suppressive axis by targeting genes associated with proliferation and EMT, such as BCL2, CCND1, and VEGFA [50,51,52,53,54]. The inverse association with *FAM171A2* indicates that the loss of these miRNAs or a reduction in binding efficiency may alleviate translational repression, thereby contributing to the accumulation of *FAM171A2* mRNA despite limited protein production. Conversely, the lncRNA–mRNA correlations demonstrated dual-directional regulation: BACE1-AS exhibited a positive relationship with FAM171A2, potentially functioning as a ceRNA that sequesters inhibitory miRNAs. In contrast, PVT1 and UCA1 showed strong negative correlations, aligning with the lncRNA-mediated recruitment of repressive miRNA–RISC complexes. In endometrial carcinoma, a distinct regulatory pattern was observed, with LINC00582, LINC-ROR, MEG3, NEAT1, and SNHG12 displaying significant negative correlations. Notably, PVT1 has been implicated in ovarian cancer tumorigenesis and proposed as a promising diagnostic and therapeutic target [55], The negative correlation with FAM171A2 may indicate a compensatory or antagonistic function within tumor progression pathways. The long non-coding RNA UCA1 has been demonstrated to actively contribute to paclitaxel (PTX) resistance in ovarian cancer, where its persistent expression is crucial for sustaining the chemoresistant phenotype [56]. The present findings underscore the potential of UCA1 as a therapeutic target for overcoming PTX resistance. Our analysis reveals an inverse correlation between UCA1 and *FAM171A2* expression, suggesting that UCA1-mediated oncogenic signaling may suppress *FAM171A2*-associated regulatory pathways, thereby enhancing drug resistance mechanisms in ovarian cancer cells. Notably, MEG3 and NEAT1 are well-established tumor-suppressive lncRNAs known to regulate the PI3K/AKT, Wnt/β-catenin, and p53 signaling pathways [57,58,59,60,61,62]. The inverse association with FAM171A2 suggests that the repression of FAM171A2 may constitute a component of an anti-oncogenic response in UCEC. Collectively, these findings indicate the presence of tumor-type-specific ceRNA networks, wherein distinct sets of miRNAs and lncRNAs modulate *FAM171A2* expression. This regulatory layer may account for the observed RNA–protein discrepancy and supports the notion that *FAM171A2* expression is primarily governed by post-transcriptional control mechanisms rather than solely by transcriptional activation. Further experimental validation, such as dual-luciferase assays and loss-/gain-of-function studies, will be crucial to confirm these ceRNA interactions and elucidate their biological implications in gynecologic malignancies.

Although the protein level of FAM171A2 was low and did not correlate with OS in OV or UCEC, this finding does not preclude a functional role. Increasing evidence indicates that certain genes exert biological influence through non-coding or regulatory RNA functions, localized translation, or EV-mediated signaling rather than through abundant protein production. The consistent upregulation of *FAM171A2* transcripts, coupled with its strong association with post-transcriptional regulators (miR-15/16/195/497 family, UCA1, PVT1, LINC00582, MEG3, and NEAT1), suggests that its biological significance resides in its participation in ceRNA and EV-associated networks that modulate gene expression, cellular adhesion, and microenvironmental interactions. Thus, *FAM171A2* may act as a transcript-level regulatory node, influencing gynecologic tumor behavior without requiring high protein expression. Importantly, this study provides the first integrated, multi-dataset analysis of FAM171A2 in ovarian and endometrial cancer, establishing a foundation for future mechanistic and translational investigations.

### Limitations and Future Directions

This study is based on integrated bioinformatic analyses and therefore carries inherent limitations. Computational predictions are influenced by dataset heterogeneity, batch effects, tissue sampling variation, and algorithm-specific inference biases. While we utilized cross-platform validation across TCGA, GEO, HPA, KMplot, UALCAN, and TNMplot to increase robustness, the proposed molecular interactions and functional associations of *FAM171A2* cannot be considered mechanistic evidence without experimental validation. Future work should include loss- and gain-of-function assays, CRISPR/Cas9-mediated knockout or overexpression in OV/UCEC cell models, and reporter-based miRNA interaction assays, followed by in vivo xenograft studies to clarify functional roles. Moreover, integration with single-cell and spatial transcriptomics may help resolve FAM171A2-mediated communication within tumor, immune, and stromal compartments. Such validation will be essential to translate these computational observations into clinically actionable insights. Since no significant association with survival was observed in OV or UCEC, and therefore *FAM171A2* cannot currently be considered a prognostic or therapeutic biomarker.

## 4. Materials and Methods

TCGA-OV and TCGA-UCEC RNA-seq datasets were accessed through GEPIA2, UALCAN, TIMER2 and TNMplot and are presented as transcript-per-million (TPM) normalized values. GEO microarray datasets (GSE17025, GSE63678, GSE14407, GSE18520, GSE36368) were downloaded from GEO and processed using the limma package (version 3.58.1) with RMA normalization followed by log2 transformation. All differential expression analyses were performed with FDR correction using the Benjamini–Hochberg method. We have now added Supplementary Table S1, which summarizes all datasets used in the study, including the cancer type, platform, sample size (tumor vs. normal), accession numbers, and preprocessing methods.

### 4.1. Gene Expression Using the GEPIA2 Web Server

Gene expression analyses were conducted using the GEPIA2 (Gene Expression Profiling Interactive Analysis 2) web tool (http://gepia2.cancer-pku.cn/, accessed on 15 September 2025). GEPIA2 is based on reprocessed RNA-seq data derived from the TCGA (The Cancer Genome Atlas) and GTEx (Genotype-Tissue Expression) projects, providing a comprehensive platform for transcriptomic exploration. The tool enables interactive analyses including tumor versus normal expression comparison, differential expression profiling, survival analysis, correlation evaluation, and isoform-level investigation. All analyses were performed using the default parameters of GEPIA2. For differential expression, a |log_2_FC| threshold was applied, and results with *p* < 0.05 were considered statistically significant [63]. Protein expression and localization data of FAM171A2 and related genes were obtained from the Human Protein Atlas (HPA; https://www.proteinatlas.org/ accessed on 15 September 2025) database. The HPA provides immunohistochemistry (IHC)-based protein expression profiles across normal and tumor tissues, as well as transcriptomic data derived from RNA sequencing (RNA-seq). In this study, the “Pathology Atlas” and “Tissue Atlas” modules were used to evaluate the expression of candidate genes in gynecological cancers (UCEC and OV) and corresponding normal tissues. Immunohistochemical staining patterns, intensity, and cell type specificity were systematically analyzed, and RNA expression levels were reported as normalized transcripts per million (TPM). Data extraction and interpretation followed the standardized HPA guidelines [11].

### 4.2. Normal and Tumor Comparisons via TNMplot

Gene expression analyses were performed using the TNMplot.com web tool (http://tnmplot.com, accessed on 15 September 2025), which integrates uniformly processed datasets from The Cancer Genome Atlas (TCGA), Genotype-Tissue Expression (GTEx), and Gene Expression Omnibus (GEO) repositories. The platform provides a comprehensive framework to compare transcriptomic profiles across normal, tumor, and metastatic tissues. All available tumor types included in TNMplot were analyzed to systematically evaluate the expression levels of the selected genes across diverse malignancies. For each cancer entity, paired or unpaired tumor and corresponding normal tissue data were retrieved, and where available, metastatic samples were also incorporated into the analysis. For normal vs. tumor comparisons (OV and UCEC), the Mann–Whitney U test was employed. A threshold of *p* < 0.05 was regarded as statistically significant [64]. Differential gene expression analysis between tumor and normal tissues was performed using the TIMER2.0 web server (Tumor Immune Estimation Resource; https://compbio.cn/timer2/ accessed on 15 September 2025 [65]. TIMER2 integrates TCGA datasets and provides Wilcoxon rank-sum test–based comparisons across multiple cancer types. Boxplots representing FAM171A2 expression (log2 TPM) in tumor and matched normal samples were generated, and statistical significance was indicated as * *p* < 0.05, ** *p* < 0.01, and *** *p* < 0.001.

### 4.3. UALCAN-Based Expression Analysis in OV and UCEC

UALCAN (http://ualcan.path.uab.edu, accessed on 15 September 2025) was employed to evaluate the mRNA expression of the selected genes in ovarian cancer (OV) and uterine corpus endometrial carcinoma (UCEC) using TCGA datasets. Expression differences between tumor and normal tissues, as well as across clinicopathological parameters such as stage, grade, and patient demographics, were analyzed. Statistical comparisons were conducted using Student’s *t*-test, with *p* < 0.05 considered significant [66,67].

### 4.4. Kaplan–Meier Plotter Workflow for Gene-Expression–Survival Analyses

Survival analyses were performed using the Kaplan–Meier Plotter (KMplot; https://kmplot.com/analysis, accessed on 15 September 2025). For the selected tumor module, patients with complete survival data were included. The primary endpoint was overall survival (OS); progression-/disease-free survival (PFS/DFS) was evaluated when available. Gene expression values (RNA-seq or microarray) were taken as provided by KMplot; when multiple probes represented a gene in microarray datasets, the JetSet algorithm was used to select the optimal probe. Kaplan–Meier curves were generated and groups compared with two-sided log-rank tests; hazard ratios (HRs) with 95% confidence intervals were estimated using univariable Cox models. Statistical significance was set at *p* < 0.05 [28,68,69].

### 4.5. Prediction of FAM171A2 miRNA Interactions Using TargetScan 8.0

Computational miRNA–target predictions were obtained TargetScanHuman Release 8.0 (https://www.targetscan.org, accessed on 15 September 2025) (accessed on current versions). TargetScan 8.0 identifies putative mammalian target sites based on 8mer/7mer/6mer seed matches and ranks candidates using the context++ family of metrics; in version 8.0 the ranking is further informed by a biochemical model of targeting efficacy. Family-level predictions were downloaded from TargetScanHuman 8.0. For each gene we recorded the cumulative weighted context++ score (CWCS) and, where available, the probability of conserved targeting (PCT). Because more negative CWCS values predict stronger repression, ranking and interpretation were based on these metrics [70].

### 4.6. STRING Database-Based Analysis of FAM171A2 Interacting Proteins

To investigate the interaction landscape of FAM171A2, protein–protein interaction (PPI) networks were constructed using both the STRING database (version 12.0; https://string-db.org/, accessed on 15 September 2025). The STRING analysis included both experimentally validated and predicted interactions derived from curated databases, high-throughput experiments, co-expression data, gene neighborhood, gene fusion, co-occurrence, and text mining. The interaction score threshold was set to 0.4 (medium confidence) to ensure balanced sensitivity and specificity. Interactions with a score below this threshold were excluded. The resulting PPI network of FAM171A2 and its interacting partners was visualized and exported for further topological analysis, including the identification of hub proteins and functional modules. These data were subsequently integrated into downstream bioinformatics analyses to evaluate the potential biological role of FAM171A2 in tumor development and progression [71].

### 4.7. Gene Expression Data Acquisition from GEO

Gene expression datasets were retrieved from the Gene Expression Omnibus (GEO) database (https://www.ncbi.nlm.nih.gov/geo/, accessed on 15 September 2025) [72]. For UCEC, we analyzed GSE17025 (Affymetrix Human Genome U133 Plus 2.0 Array) [73,74] and GSE7305 (Affymetrix Human Gene 1.0 ST Array) [75]. For ovarian cancer, we included GSE14407 (Affymetrix Human Genome U133A Array) [76], GSE18520 Affymetrix U133A [77], and GSE36668Affymetrix U133 Plus 2.0 Array [78].

Raw or processed data (MINiML/SOFT) were downloaded and preprocessed according to each platform’s annotation. Differential expression analyses were performed using the GEO2R web tool/https://www.ncbi.nlm.nih.gov/geo/geo2r/ accessed on 15 September 2025), which applies the limma (Linear Models for Microarray Data) package in R. *p*-values were adjusted for multiple testing using the Benjamini–Hochberg false discovery rate (FDR) method. Genes with |log2FC| > 1 and adjusted *p* < 0.05 were considered significantly differentially expressed. Dataset details including accession numbers, sample counts, and platforms are summarized in Supplementary Tables S2–S6.

### 4.8. Analysis of FAM171A2–miRNA and lncRNA Interactions Using the ENCORI Database

Putative lncRNA-miRNA–mRNA interactions involving FAM171A2 were analyzed using the ENCORI (The Encyclopedia of RNA Interactomes; formerly StarBase v3.0) database (https://starbase.sysu.edu.cn/, accessed on 15 September 2025) [79]. ENCORI integrates large-scale CLIP-Seq, degradome-Seq, and RNA-RNA interaction data from TCGA, ENCODE, and other high-throughput transcriptomic projects, enabling systematic investigation of post-transcriptional regulatory networks. For this study, the lncRNA -miRNA–mRNA module was queried to identify candidate miRNAs potentially targeting FAM171A2. The filtering parameters were set as CLIP-Data ≥ 3, Degradome-Data ≥ 1, and pan-Cancer ≥ 1, ensuring high-confidence interactions supported by multiple experimental datasets. Expression correlations between *FAM171A2* and the predicted miRNAs were analyzed in uterine corpus endometrial carcinoma (UCEC) and ovarian serous cystadenocarcinoma (OV) using TCGA-derived RNA-Seq data incorporated in ENCORI. Spearman’s correlation coefficients (ρ) were used to determine the strength and direction of associations between *FAM171A2* mRNA and miRNA expression levels.

### 4.9. Statistical Analysis

To ensure methodological transparency and consistency across platforms, all transcriptomic analyses were conducted using harmonized statistical parameters. For RNA-seq datasets obtained through GEPIA2, UALCAN, TIMER2, and TNMplot, gene expression values were analyzed using log2(TPM + 1) normalization and the Wilcoxon rank-sum test, with multiple testing controlled by the Benjamini–Hochberg false discovery rate (FDR) correction. Microarray-based GEO datasets (GSE17025, GSE63678, GSE14407, GSE18520, GSE36368) were processed using the limma package with RMA normalization followed by log2 transformation, and FDR-adjusted *p*-values were reported. For differential expression visualization in GEPIA2, platform defaults were applied (|log2FC| ≥ 1, *p* < 0.05). Protein interaction networks were constructed using STRING (v12) with a combined confidence score ≥ 0.70, maximum interactors = 50, and evidence restricted to experimental, curated database, and co-expression sources, with disconnected nodes removed. This harmonized workflow minimizes dataset-specific bias and enhances reproducibility across independent platforms.

## 5. Conclusions

In summary, this study presents the inaugural integrative characterization of *FAM171A2* across gynecologic malignancies, revealing tumor-type-specific expression patterns and distinct post-transcriptional regulatory signatures. While *FAM171A2* mRNA levels are elevated in ovarian carcinoma and modestly altered in endometrial carcinoma, protein expression remains low, indicating a discrepancy between transcription and translation. Correlation analyses identified a network of miRNAs and lncRNAs—including the miR-15/16/195/497 family, BACE1-AS, PVT1, UCA1, LINC00582, MEG3, NEAT1, and SNHG12—that may fine-tune FAM171A2 abundance in a context-dependent manner. These findings underscore post-transcriptional regulation as a predominant mechanism governing *FAM171A2* expression. Although *FAM171A2* was not independently prognostic for overall survival, its association with neuronal and immune-related gene modules suggests potential involvement in cell–cell communication, extracellular vesicle biology, and tumor microenvironmental remodeling. Further functional validation through cell-based and animal models will be critical to elucidate its mechanistic role and assess whether *FAM171A2* or its regulatory RNA network can serve as a novel biomarker or therapeutic target in gynecologic cancers.

## Data Availability

Data is contained within the article and Supplementary Materials. Further inquiries can be directed to the corresponding author.

## Supplementary Materials

The following supporting information can be downloaded at: https://www.mdpi.com/article/10.3390/ijms262211126/s1.

## Abbreviations

The following abbreviations are used in this manuscript:

AUC	Area Under the Curve
BMI	Body Mass Index
ceRNA	Competing Endogenous RNA
CI	Confidence Interval
CLIP-Seq	Cross-Linking Immunoprecipitation Sequencing
ECM	Extracellular Matrix
ENCORI	Encyclopedia of RNA Interactomes
EMT	Epithelial–Mesenchymal Transition
EV	Extracellular Vesicle
FC	Fold Change
FDR	False Discovery Rate
GEPIA2	Gene Expression Profiling Interactive Analysis 2
GEO	Gene Expression Omnibus
GTEx	Genotype-Tissue Expression
HGSC	High-Grade Serous Carcinoma
HPA	Human Protein Atlas
HR	Hazard Ratio
IHC	Immunohistochemistry
KMplot	Kaplan–Meier Plotter
lncRNA	Long Non-Coding RNA
miRNA	MicroRNA
mRNA	Messenger RNA
NF-κB	Nuclear Factor kappa-light-chain-enhancer of activated B cells
OV	Ovarian Cancer
PPI	Protein–Protein Interaction
PTX	Paclitaxel
RISC	RNA-Induced Silencing Complex
RNA-seq	RNA Sequencing
STRING	Search Tool for the Retrieval of Interacting Genes/Proteins
TCGA	The Cancer Genome Atlas
TIMER2	Tumor Immune Estimation Resource 2
TNMplot	Tumor–Normal–Metastatic Plotter
TP53	Tumor Protein 53
TPM	Transcripts Per Million
UCEC	Uterine Corpus Endometrial Carcinoma
UALCAN	University of Alabama at Birmingham Cancer Data Analysis Portal
VEGFA	Vascular Endothelial Growth Factor A
ZNF696	Zinc Finger Protein 696

## References

[1] Jiang Y., Xu Y., He J., Sui L., Li T., Xia N., Yao Q. (2025). Uncovering potential targets for antibody-drug conjugates in the treatment of gynecologic malignancies. Front. Pharmacol..

[2] Bodriagova O., Previs R.A., Gaba L., Shankar A., Vidal L., Saini K.S. (2023). Recent Advances in Gynecological Malignancies: Focus on ASCO 2023. Oncol. Ther..

[3] Aravantinou-Fatorou A., Georgakopoulou V.E., Dimopoulos M.A., Liontos M. (2025). Precision medicine in gynecological cancer (Review). Biomed. Rep..

[4] Rasila T., Saavalainen O., Attalla H., Lankila P., Haglund C., Hölttä E., Andersson L.C. (2019). Astroprincin (FAM171A1, C10orf38): A Regulator of Human Cell Shape and Invasive Growth. Am. J. Pathol..

[5] Wahab A., Almangush A., Andersson L.C., Nieminen P., Salo T. (2020). Impact of Astroprincin (FAM171A1) Expression in Oral Tongue Cancer. Front. Oral Health.

[6] Bao C., Lu Y., Chen J., Chen D., Lou W., Ding B., Xu L., Fan W. (2019). Exploring specific prognostic biomarkers in triple-negative breast cancer. Cell Death Dis..

[7] Sanawar R., Mohan Dan V., Santhoshkumar T.R., Kumar R., Pillai M.R. (2019). Estrogen receptor-α regulation of microRNA-590 targets FAM171A1—A modifier of breast cancer invasiveness. Oncogenesis.

[8] Deng H., Xu X., Zhang Y., Li Y. (2025). The complex role and molecular mechanism of family with sequence similarity genes in cancer: A comprehensive review. Discov. Oncol..

[9] UniProt UniProt n.d. https://www.uniprot.org/uniprotkb/Q8N8K9/entry.

[10] FAM171A2 Protein Expression Summary—The Human Protein Atlas n.d. https://www.proteinatlas.org/ENSG00000161682-FAM171A2.

[11] Uhlén M., Fagerberg L., Hallström B.M., Lindskog C., Oksvold P., Mardinoglu A., Sivertsson Å., Kampf C., Sjöstedt E., Asplund A. (2015). Tissue-based map of the human proteome. Science.

[12] Kaprio T., Lindström A.M., Rasila T., Saavalainen O., Beilmann-Lehtonen I., Mustonen H., Haglund C., Andersson L.C. (2021). Elevated tumor expression of Astroprincin (FAM171A1) is an independent marker of poor prognosis in colon cancer. BMC Gastroenterol..

[13] Xu W., Han S.-D., Zhang C., Li J.-Q., Wang Y.-J., Tan C.-C., Li H.-Q., Dong Q., Mei C., Tan L. (2020). The FAM171A2 gene is a key regulator of progranulin expression and modifies the risk of multiple neurodegenerative diseases. Sci. Adv..

[14] Hanahan D. (2022). Hallmarks of Cancer: New Dimensions. Cancer Discov..

[15] Kośla K., Orzechowska M., Jędroszka D., Baryła I., Bednarek A.K., Płuciennik E. (2019). A Novel Set of WNT Pathway Effectors as a Predictive Marker of Uterine Corpus Endometrial Carcinoma–Study Based on Weighted Co-expression Matrices. Front. Oncol..

[16] Chen H.-X., Xu X.-X., Tan B.-Z., Zhang Z., Zhou X.-D. (2017). MicroRNA-29b Inhibits Angiogenesis by Targeting VEGFA through the MAPK/ERK and PI3K/Akt Signaling Pathways in Endometrial Carcinoma. Cell. Physiol. Biochem. Int. J. Exp. Cell. Physiol. Biochem. Pharmacol..

[17] Diéguez-Martínez N., Espinosa-Gil S., Yoldi G., Megías-Roda E., Bolinaga-Ayala I., Viñas-Casas M., Gorgisen G., Domingo-Ortí I., Pérez-Montoyo H., Bayascas J.R. (2022). The ERK5/NF-κB signaling pathway targets endometrial cancer proliferation and survival. Cell. Mol. Life Sci..

[18] Wang Y., Shan J., Zhang L., Wang R., Wu M.-Y., Li H.-M., Xu H.-M. (2025). The role of FAM171A2-GRN-NF-κB pathway in TBBPA induced oxidative stress and inflammatory response in mouse-derived hippocampal neuronal HT22 cells. Ecotoxicol. Environ. Saf..

[19] Cho A., Howell V.M., Colvin E.K. (2015). The Extracellular Matrix in Epithelial Ovarian Cancer—A Piece of a Puzzle. Front. Oncol..

[20] Loret N., Denys H., Tummers P., Berx G. (2019). The Role of Epithelial-to-Mesenchymal Plasticity in Ovarian Cancer Progression and Therapy Resistance. Cancers.

[21] Fang D., Chen H., Zhu J.Y., Wang W., Teng Y., Ding H., Jing Q., Su S., Huang S. (2017). Epithelial–mesenchymal transition of ovarian cancer cells is sustained by Rac1 through simultaneous activation of MEK1/2 and Src signaling pathways. Oncogene.

[22] Liu Y., Beyer A., Aebersold R. (2016). On the Dependency of Cellular Protein Levels on mRNA Abundance. Cell.

[23] Vogel C., Marcotte E.M. (2012). Insights into the regulation of protein abundance from proteomic and transcriptomic analyses. Nat. Rev. Genet..

[24] Wang X., Wang J., Ma H., Zhang J., Zhou X. (2012). Downregulation of miR-195 correlates with lymph node metastasis and poor prognosis in colorectal cancer. Med. Oncol..

[25] Zhao X., Dai L., Yue Q., Wang H., Wang X.U., Li Y., Chen R. (2019). MiR-195 inhibits migration, invasion and epithelial-mesenchymal transition (EMT) of endometrial carcinoma cells by targeting SOX4. J. Biosci..

[26] Zare E., Yaghoubi S.M., Khoshnazar M., Jafari Dargahlou S., Machhar J.S., Zheng Z., Duijf P.H.G., Mansoori B. (2025). MicroRNAs in Cancer Immunology: Master Regulators of the Tumor Microenvironment and Immune Evasion, with Therapeutic Potential. Cancers.

[27] Yang Y., Jiang Y., Wan Y., Zhang L., Qiu J., Zhou S., Cheng W. (2016). UCA1 functions as a competing endogenous RNA to suppress epithelial ovarian cancer metastasis. Tumor Biol..

[28] Gao Y.L., Zhao Z.S., Zhang M.Y., Han L.J., Dong Y.J., Xu B. (2017). Long Noncoding RNA PVT1 Facilitates Cervical Cancer Progression via Negative Regulating of miR-424. Oncol. Res..

[29] Salmena L., Poliseno L., Tay Y., Kats L., Pandolfi P.P. (2011). A ceRNA hypothesis: The Rosetta Stone of a hidden RNA language?. Cell.

[30] Liu Y.J., Wang C. (2023). A review of the regulatory mechanisms of extracellular vesicles-mediated intercellular communication. Cell Commun. Signal..

[31] Cancer Genome Atlas Research Network (2011). Integrated genomic analyses of ovarian carcinoma. Nature.

[32] Polajžer S., Černe K. (2025). Precision Medicine in High-Grade Serous Ovarian Cancer: Targeted Therapies and the Challenge of Chemoresistance. Int. J. Mol. Sci..

[33] Győrffy B., Surowiak P., Budczies J., Lánczky A. (2013). Online Survival Analysis Software to Assess the Prognostic Value of Biomarkers Using Transcriptomic Data in Non-Small-Cell Lung Cancer. PLoS ONE.

[34] Van De Vijver M.J., He Y.D., Van’t Veer L.J., Dai H., Hart A.A., Voskuil D.W., Schreiber G.J., Peterse J.L., Roberts C., Marton M.J. (2002). A Gene-Expression Signature as a Predictor of Survival in Breast Cancer. N. Engl. J. Med..

[35] Qian Y., Daza J., Itzel T., Betge J., Zhan T., Marmé F., Teufel A. (2021). Prognostic Cancer Gene Expression Signatures: Current Status and Challenges. Cells.

[36] Xu X., Yin F., Guo M., Gan G., Lin G., Wen C., Wang J., Song P., Wang J., Qi Z.Q. (2023). Quantitative proteomic analysis of exosomes from umbilical cord mesenchymal stem cells and rat bone marrow stem cells. Proteomics.

[37] Liang B., Peng P., Chen S., Li L., Zhang M., Cao D., Yang J., Li H., Gui T., Li X. (2013). Characterization and proteomic analysis of ovarian cancer-derived exosomes. J. Proteom..

[38] Ghasemzadeh T., Rajabi A., MalekAbbaslou E., Najari P., Akbarzadeh S., Tayefeh-Gholami S., Teimourian S., Hosseinpourfeizi M., Safaralizadeh R. (2023). Evaluation of the expression of the long non-coding RNAs, LOWEG and MINCR, and their clinical significance in human gastric cancer. Egypt. J. Med. Hum. Genet..

[39] Wu J., Liu G., An K., Shi L. (2022). NPTX1 inhibits pancreatic cancer cell proliferation and migration and enhances chemotherapy sensitivity by targeting RBM10. Oncol. Lett..

[40] Peng X., Pan K., Zhao W., Zhang J., Yuan S., Wen X., Zhou W., Yu Z. (2018). NPTX1 inhibits colon cancer cell proliferation through down-regulating cyclin A2 and CDK2 expression. Cell Biol. Int..

[41] Maino B., Ciotti M.T., Calissano P., Cavallaro S. (2014). Transcriptional Analysis of Apoptotic Cerebellar Granule Neurons Following Rescue by Gastric Inhibitory Polypeptide. Int. J. Mol. Sci..

[42] Apóstolo N., Smukowski S.N., Vanderlinden J., Condomitti G., Rybakin V., Ten Bos J., Trobiani L., Portegies S., Vennekens K.M., Gounko N.V. (2020). Synapse type-specific proteomic dissection identifies IgSF8 as a hippocampal CA3 microcircuit organizer. Nat. Commun..

[43] Vescio M., Paracchini L., Beltrame L., D’Incalci M., Marchini S., Pattini L. (2023). Modulation of gene expression associated with copy number variation identifies key regulatory programs in high-grade serous ovarian carcinoma. Adv. Cancer Biol. Metastasis.

[44] Toulza E., Mattiuzzo N.R., Galliano M.F., Jonca N., Dossat C., Jacob D., De Daruvar A., Wincker P., Serre G., Guerrin M. (2007). Large-scale identification of human genes implicated in epidermal barrier function. Genome Biol..

[45] Wu K.M., Xu Q.H., Liu Y.Q., Feng Y.-W., Han S.-D., Zhang Y.-R., Chen S.-D., Guo Y., Wu B.-S., Ma L.-Z. (2025). Neuronal FAM171A2 mediates α-synuclein fibril uptake and drives Parkinson’s disease. Science.

[46] Magnon C., Hall S.J., Lin J., Xue X., Gerber L., Freedland S.J., Frenette P.S. (2013). Autonomic nerve development contributes to prostate cancer progression. Science.

[47] Zahalka A.H., Frenette P.S. (2020). Nerves in cancer. Nat. Rev. Cancer.

[48] Kalluri R., LeBleu V.S. (2020). The biology, function, and biomedical applications of exosomes. Science.

[49] Wortzel I., Dror S., Kenific C.M., Lyden D. (2019). Exosome-Mediated Metastasis: Communication from a Distance. Dev. Cell.

[50] Schwarzenbach H. (2016). Clinical significance of miR-15 and miR-16 in ovarian cancer. Transl. Cancer Res..

[51] Cimmino A., Calin G.A., Fabbri M., Iorio M.V., Ferracin M., Shimizu M., Wojcik S.E., Aqeilan R.I., Zupo S., Dono M. (2005). miR-15 and miR-16 induce apoptosis by targeting BCL2. Proc. Natl. Acad. Sci. USA.

[52] Zhang S.-Y., Lu Z.-M., Lin Y.-F., Chen L.-S., Luo X.-N., Song X.-H., Chen S.-H., Wu Y.-L. (2016). miR-144-3p, a tumor suppressive microRNA targeting ETS-1 in laryngeal squamous cell carcinoma. Oncotarget.

[53] Yan J.-J., Zhang Y.-N., Liao J.-Z., Ke K., Chang Y., Li P.-Y., Wang M., Lin J.-S., He X.-X. (2015). MiR-497 suppresses angiogenesis and metastasis of hepatocellular carcinoma by inhibiting VEGFA and AEG-1. Oncotarget.

[54] Luo G., He K., Xia Z., Liu S., Liu H., Xiang G. (2020). Regulation of microRNA-497 expression in human cancer. Oncol. Lett..

[55] Chen Y., Du H., Bao L., Liu W. (2018). LncRNA PVT1 promotes ovarian cancer progression by silencing miR-214. Cancer Biol. Med..

[56] Li Z.-Y., Wang X.-L., Dang Y., Zhu X.-Z., Zhang Y.-H., Cai B.-X., Zheng L. (2020). Long non-coding RNA UCA1 promotes the progression of paclitaxel resistance in ovarian cancer by regulating the miR-654-5p/SIK2 axis. Eur. Rev..

[57] Tang Z.-L., Zhang K., Lv S.-C., Xu G.-W., Zhang J.-F., Jia H.-Y. (2021). LncRNA MEG3 suppresses PI3K/AKT/mTOR signalling pathway to enhance autophagy and inhibit inflammation in TNF-α-treated keratinocytes and psoriatic mice. Cytokine.

[58] Pu Z., Wu L., Guo Y., Li G., Xiang M., Liu L., Zhan H., Zhou X., Tan H. (2019). LncRNA MEG3 contributes to adenosine-induced cytotoxicity in hepatoma HepG2 cells by downregulated ILF3 and autophagy inhibition via regulation PI3K-AKT-mTOR and beclin-1 signaling pathway. J. Cell. Biochem..

[59] Yang Y., Yan X., Li X., Ma Y., Goel A. (2021). Long non-coding RNAs in colorectal cancer: Novel oncogenic mechanisms and promising clinical applications. Cancer Lett..

[60] Zhou H., Wang Y., Liu Z., Zhang Z., Xiong L., Wen Y. (2022). Recent advances of NEAT1-miRNA interactions in cancer. Acta Biochim. Biophys. Sin..

[61] Adriaens C., Standaert L., Barra J., Latil M., Verfaillie A., Kalev P., Boeckx B., Wijnhoven P.W.G., Radaelli E., Vermi W. (2016). p53 induces formation of NEAT1 lncRNA-containing paraspeckles that modulate replication stress response and chemosensitivity. Nat. Med..

[62] Wu Y.-C., Ku M.-H., Huang W.-L., Dias L., Chen C.-W. (2025). NEAT1 in Ovarian Cancer: A Key Regulator of Tumor Progression, Follicular Fluid Dynamics, and Therapeutic Resistance. Anticancer Res..

[63] Tang Z., Kang B., Li C., Chen T., Zhang Z. (2019). GEPIA2: An enhanced web server for large-scale expression profiling and interactive analysis. Nucleic Acids Res..

[64] Bartha Á., Győrffy B. (2021). TNMplot.com: A Web Tool for the Comparison of Gene Expression in Normal, Tumor and Metastatic Tissues. Int. J. Mol. Sci..

[65] Li T., Fu J., Zeng Z., Cohen D., Li J., Chen Q., Li B., Liu X.S. (2020). TIMER2.0 for analysis of tumor-infiltrating immune cells. Nucleic Acids Res..

[66] Chandrashekar D.S., Bashel B., Balasubramanya S.A.H., Creighton C.J., Ponce-Rodriguez I., Chakravarthi B.V.S.K., Varambally S. (2017). UALCAN: A Portal for Facilitating Tumor Subgroup Gene Expression and Survival Analyses. Neoplasia.

[67] Chandrashekar D.S., Karthikeyan S.K., Korla P.K., Patel H., Shovon A.R., Athar M., Netto G.J., Qin Z.S., Kumar S., Manne U. (2022). UALCAN: An update to the integrated cancer data analysis platform. Neoplasia.

[68] Lánczky A., Győrffy B. (2021). Web-Based Survival Analysis Tool Tailored for Medical Research (KMplot): Development and Implementation. J. Med. Internet Res..

[69] Li Q., Birkbak N.J., Gyorffy B., Szallasi Z., Eklund A.C. (2011). Jetset: Selecting the optimal microarray probe set to represent a gene. BMC Bioinform..

[70] McGeary S.E., Lin K.S., Shi C.Y., Pham T.M., Bisaria N., Kelley G.M., Bartel D.P. (2019). The biochemical basis of microRNA targeting efficacy. Science.

[71] Szklarczyk D., Gable A.L., Lyon D., Junge A., Wyder S., Huerta-Cepas J., Simonovic M., Doncheva N.T., Morris J.H., Bork P. (2019). STRING v11: Protein–protein association networks with increased coverage, supporting functional discovery in genome-wide experimental datasets. Nucleic Acids Res..

[72] Edgar R., Domrachev M., Lash A.E. (2002). Gene Expression Omnibus: NCBI gene expression and hybridization array data repository. Nucleic Acids Res..

[73] Day R.S., McDade K.K., Chandran U.R., Lisovich A., Conrads T.P., Hood B.L., Kolli V.K., Kirchner D., Litzi T., Maxwell G.L. (2011). Identifier mapping performance for integrating transcriptomics and proteomics experimental results. BMC Bioinform..

[74] Day R.S., McDade K.K. (2013). A decision theory paradigm for evaluating identifier mapping and filtering methods using data integration. BMC Bioinform..

[75] Hever A., Roth R.B., Hevezi P., Marin M.E., Acosta J.A., Acosta H., Rojas J., Herrera R., Grigoriadis D., White E. (2007). Human endometriosis is associated with plasma cells and overexpression of B lymphocyte stimulator. Proc. Natl. Acad. Sci. USA.

[76] Bowen N.J., Walker L.D., Matyunina L.V., Logani S., Totten K.A., Benigno B.B., McDonald J.F. (2009). Gene expression profiling supports the hypothesis that human ovarian surface epithelia are multipotent and capable of serving as ovarian cancer initiating cells. BMC Med. Genom..

[77] Mok S.C., Bonome T., Vathipadiekal V., Bell A., Johnson M.E., Wong K.-K., Park D.-C., Hao K., Yip D.K.P., Donninger H. (2009). A gene signature predictive for outcome in advanced ovarian cancer identifies a survival factor: Microfibril-associated glycoprotein 2. Cancer Cell.

[78] Elgaaen B.V., Olstad O.K., Sandvik L., Odegaard E., Sauer T., Staff A.C., Gautvik K.M. (2012). ZNF385B and VEGFA are strongly differentially expressed in serous ovarian carcinomas and correlate with survival. PLoS ONE.

[79] Li J.-H., Liu S., Zhou H., Qu L.-H., Yang J.-H. (2013). starBase v2.0: Decoding miRNA-ceRNA, miRNA-ncRNA and protein–RNA interaction networks from large-scale CLIP-Seq data. Nucleic Acids Res..

