# Supplementary material for: The Emerging Role of FAM171A2 in Gynecological Malignancies: Bioinformatic Insights from UCEC and Ovarian Cancer
Source: Int J Mol Sci. 2025 Nov 18;26(22):11126. doi: 10.3390/ijms262211126 (PMC12652445; doi:10.3390/ijms262211126)
Supplement: Supplementary file 1 [file ijms-26-11126-s001.zip › Supplementary Table S1-Datasets.docx]

# Supplementary Table 1. Summary of Datasets Used in the Study

| Dataset | Cancer Type | Platform | Sample Size (Tumor / Normal) | Accession Number | Normalization Method |
| --- | --- | --- | --- | --- | --- |
| TCGA-OV | Ovarian Cancer | RNA-seq (Illumina HiSeq) | n≈379 / n≈88 (TCGA + GTEx) | TCGA-OV | TPM normalization |
| TCGA-UCEC | Endometrial Cancer | RNA-seq (Illumina HiSeq) | n≈548 / n≈35 (TCGA + GTEx) | TCGA-UCEC | TPM normalization |
| GSE17025 | Endometrial Cancer | Microarray (Affymetrix GPL570) | As per GEO metadata | GSE17025 | RMA + log2 (limma) |
| GSE63678 | Endometrial Cancer | Microarray (Illumina HumanHT-12) | As per GEO metadata | GSE63678 | RMA + log2 (limma) |
| GSE14407 | Ovarian Cancer | Microarray (Affymetrix GPL570) | 12 tumor / 12 normal | GSE14407 | RMA + log2 (limma) |
| GSE18520 | Ovarian Cancer | Microarray (Affymetrix GPL570) | 53 tumor / 10 normal | GSE18520 | RMA + log2 (limma) |
| GSE36368 | Ovarian Cancer | Microarray (Illumina humanRef-8) | As per GEO metadata | GSE36368 | RMA + log2 (limma) |
